# Supplementary material for: Free Radicals and ROS Induce Protein Denaturation by UV Photostability Assay
Source: Int J Mol Sci. 2021 Jun 17;22(12):6512. doi: 10.3390/ijms22126512 (PMC8234878; doi:10.3390/ijms22126512)
Supplement: Supplementary file 1 [file ijms-22-06512-s001.zip › ijms-1248477-supplementary.pdf]

Free Radicals and ROS Induce Protein Denaturation by UV Photostability Assay

Paolo Ruzza [1,*], Claudia Honisch [1,2], Rohanah Hussain [3] and Giuliano Siligardi [3,*]

[1] CNR (ICB-CNR) Padova Unit, Institute of Biomolecular Chemistry, Via F. Marzolo, 1, 35030 Padova, Italy; claudiahonisch@gmail.com
[2] Department of Chemical Sciences, University of Padua, Via F. Marzolo, 1, 35030 Padova, Italy
[3] Diamond Light Source Ltd., Harwell Science and Innovation Campus, Didcot OX11 0DE, Oxfordshire, UK; rohanah.hussain@diamond.ac.uk
* Correspondence: paolo.ruzza@unipd.it (P.R.); giuliano.siligardi@diamond.ac.uk (G.S.); Tel.: +44-(0)1235-778425 (G.S.); Fax: +39-049-827-5239 (P.R.)

SUPPLEMENTARY MATERIAL

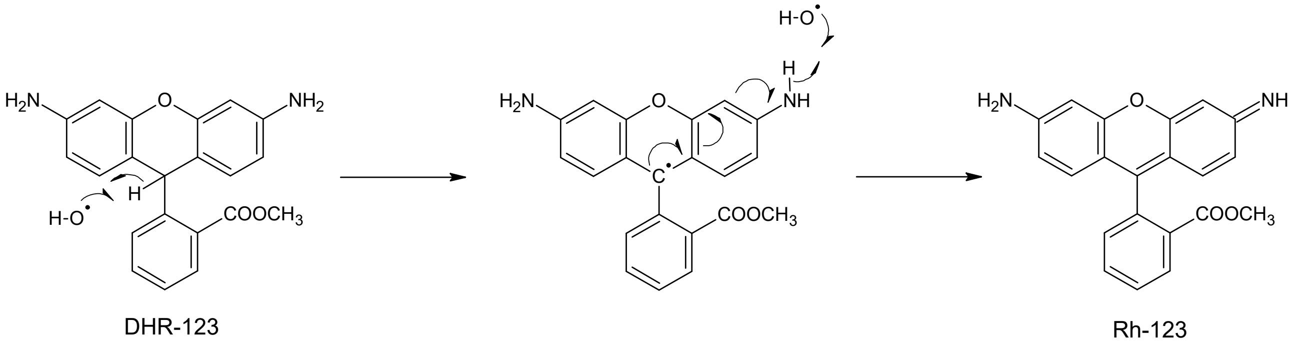

Scheme S1. *Chemical structures of Dihydrorhodamine* 123 (*DHR-123) and Rhodamine 123 (Rh-123) and proposed mechanism of reaction.*

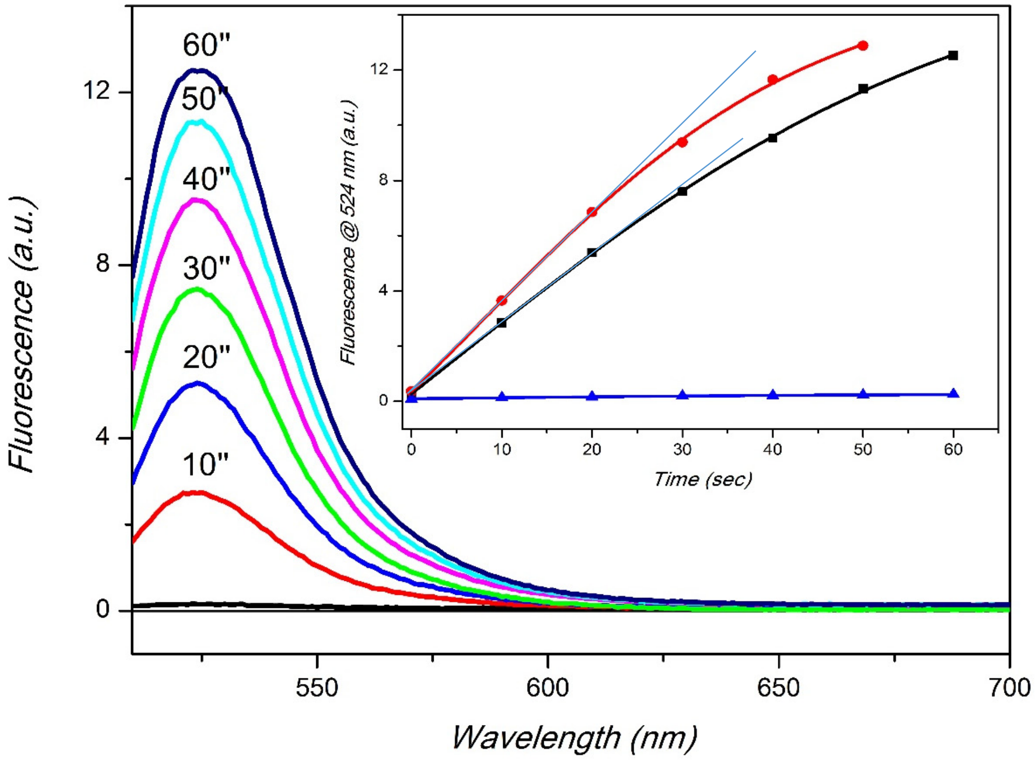

Figure S1. *Time course fluorescence emission spectra of DHR-123 (2.89 µM) irradiated with UV-C lamp. 1.5 µL of DHR-123 in DMSO (5.77 mM) was added to 3000 µL of 20 mM PBS buffer, pH 7.4. Fluorescence spectra were recorded at 25°C using a Chirascan Plus spectrometer, excitation at 505 nm, integration time 1 s, 1.0 cm cell (3000 µl), monochromator slit widths 4 nm. (Insert) Plot of the fluorescence intensity at 524 nm against reaction time of DHR-123 alone (black line) and in presence of either 0.01 mg/mL of ovalbumin (red line) or 0.1 mM ascorbic acid (blue line).*

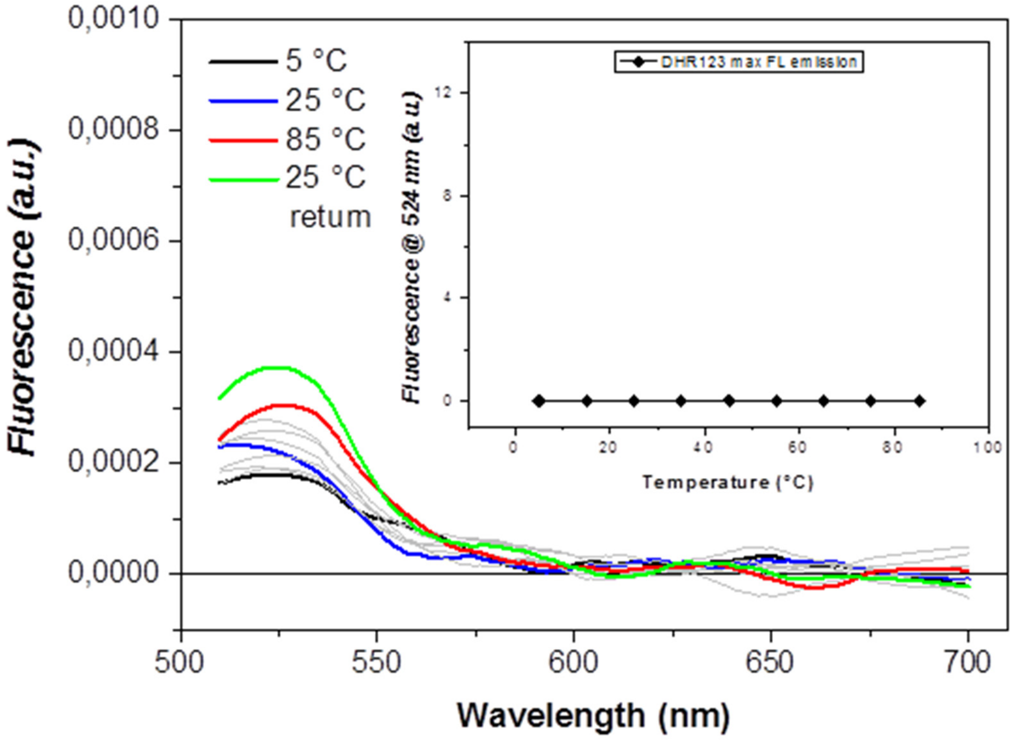

Figure S2. *Fluorescence emission spectra of DHR-123 (2.89 μM) as a function of temperature. 1.5 μL of DHR-123 in DMSO (5.77 mM) was added to 3000 μL of 20 mM PBS buffer, pH 7.4. Fluorescence spectra were recorded between 5°C and 85°C, with 10°C steps, allowing 8 minutes of equilibration, using a Chirascan Plus spectrometer, excitation at 505 nm, integration time 1 s, 1.0 cm cell (3000 μl), monochromator slit widths 4 nm. (Insert) Plot of the fluorescence intensity at 524 nm against temperature ramping.*

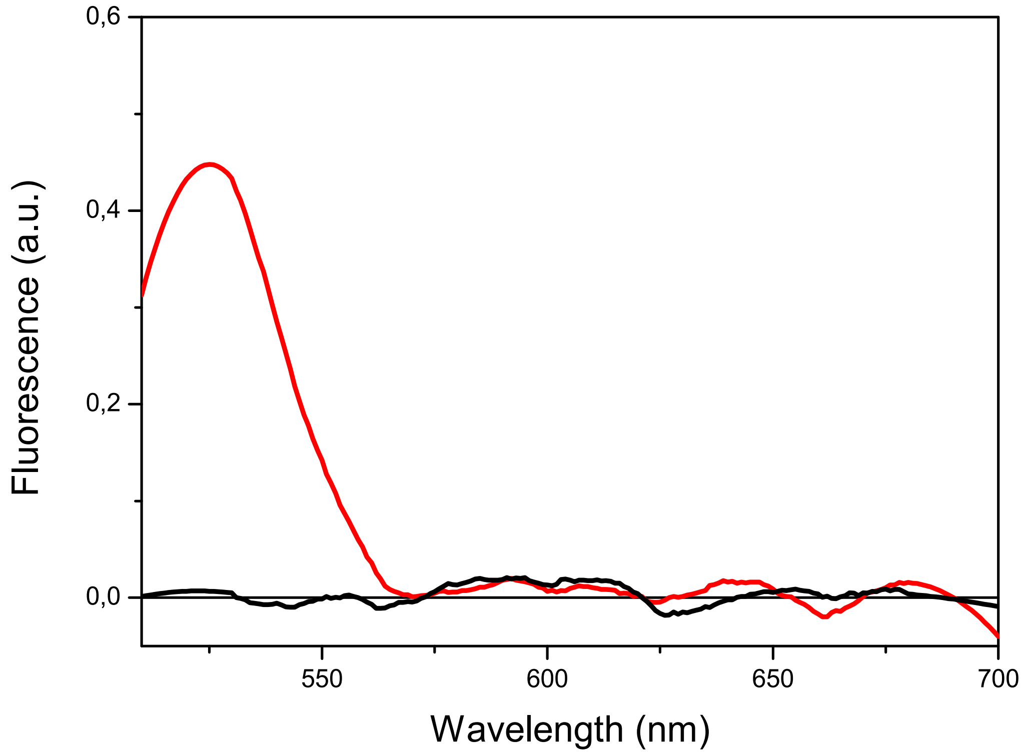

Figure S3. *Fluorescence emission spectra of DHR-123 (2.88 µM) in 20 mM PBS buffer, pH 7.4, before (black) and after (red) four consecutive repeated scans from 185 to 260 nm at the B23 beamline module end station (slit = 1 mm, integration time of 1s, 1 nm digital resolution, 39 nm/min scan speed). Ex: 505 nm*.
